# Costs and Outcomes of Increasing Access to Bariatric Surgery: Cohort Study and Cost-Effectiveness Analysis Using Electronic Health Records^☆^

**DOI:** 10.1016/j.jval.2016.08.734

**Published:** 2017-01

**Authors:** Martin C. Gulliford, Judith Charlton, Toby Prevost, Helen Booth, Alison Fildes, Mark Ashworth, Peter Littlejohns, Marcus Reddy, Omar Khan, Caroline Rudisill

**Affiliations:** 1 Department of Primary Care and Public Health Sciences, King’s College London, London, UK; 2 National Institutes for Health Research Biomedical Research Centre at Guy’s and St Thomas’ National Health Service Foundation Trust, London, UK; 3 Department of Surgery, St George’s University Hospital National Health Service Foundation Trust, London, UK; 4 Department of Social Policy, London School of Economics and Political Science, London, UK

**Keywords:** bariatric surgery, cost-effectiveness analysis, diabetes mellitus, obesity

## Abstract

**Objectives:**

To estimate costs and outcomes of increasing access to bariatric surgery in obese adults and in population subgroups of age, sex, deprivation, comorbidity, and obesity category.

**Methods:**

A cohort study was conducted using primary care electronic health records, with linked hospital utilization data, for 3,045 participants who underwent bariatric surgery and 247,537 participants who did not undergo bariatric surgery. Epidemiological analyses informed a probabilistic Markov model to compare bariatric surgery, including equal proportions with adjustable gastric banding, gastric bypass, and sleeve gastrectomy, with standard nonsurgical management of obesity. Outcomes were quality-adjusted life-years (QALYs) and net monetary benefits at a threshold of £30,000 per QALY.

**Results:**

In a UK population of 250,000 adults, there may be 7,163 people with morbid obesity including 1,406 with diabetes. The immediate cost of 1,000 bariatric surgical procedures is £9.16 million, with incremental discounted lifetime health care costs of £15.26 million (95% confidence interval £15.18–£15.36 million). Patient-years with diabetes mellitus will decrease by 8,320 (range 8,123–8,502). Incremental QALYs will increase by 2,142 (range 2,032–2,256). The estimated cost per QALY gained is £7,129 (range £6,775–£7,506). Net monetary benefits will be £49.02 million (range £45.72–£52.41 million). Estimates are similar for subgroups of age, sex, and deprivation. Bariatric surgery remains cost-effective if the procedure is twice as costly, or if intervention effect declines over time.

**Conclusions:**

Diverse obese individuals may benefit from bariatric surgery at acceptable cost. Bariatric surgery is not cost-saving, but increased health care costs are exceeded by health benefits to obese individuals.

## Introduction

The recent increase in obesity has been accompanied by a disproportionate increase in people affected by severe and morbid obesity [1]. People with morbid obesity are at increased risk of diabetes, cardiovascular diseases, and depression, leading to the development of multiple morbidities at young ages and heightened risk of cardiovascular mortality [2]. The term *bariatric surgery* refers to the use of surgical procedures for the control of body weight. It is becoming increasingly clear that bariatric surgery has important effects on obesity-related comorbidities, especially type 2 diabetes. These effects are not exclusively mediated by changes in body weight. Bariatric surgery is sometimes also referred to as *metabolic surgery*. Bariatric surgery may offer important health benefits to people with severe and morbid obesity, including reductions in body weight [3], remission of established type 2 diabetes [4], lower incidence of type 2 diabetes [5], [6] and other long-term conditions [7], as well as reduction in mortality [3], [8]. The evidence base, however, is changing. Different types of surgical procedures are evolving over time, with declining use of adjustable gastric banding and increasing use of gastric bypass and sleeve gastrectomy procedures [9]. New evidence has also emerged concerning longer term outcomes and costs [10], [11] of bariatric surgery, including effects on mortality [8], disease incidence [5], and diabetes remission [12], [13].

The role of bariatric surgery in the management of obesity remains controversial. Access to bariatric surgery is often limited by health care systems. In the United Kingdom, approximately 10,000 bariatric surgery procedures are performed annually [14], but there are more than 1 million individuals with morbid obesity who could potentially benefit from the procedure [15]. This limited access to bariatric surgery may be related to perceptions that obesity might sometimes be a “lifestyle choice” and that the cost of surgery, and the resources required to offer it more readily, might be difficult to justify. Some studies suggest that bariatric surgery may be cost-saving to health systems [16], but this is not supported by empirical studies of health care utilization after bariatric surgery [17]. A recent study concluded that “bariatric surgery does not reduce overall health care costs in the long-term” and suggested that “future studies should focus on the potential benefit of improved health and well-being of persons undergoing the procedure rather than cost-savings” [17].

The purpose of the present research was to investigate the potential population impacts of bariatric surgery used at scale for severe and morbid obesity. We aimed to evaluate the cost-effectiveness of bariatric surgery, comprising laparoscopic gastric banding, gastric bypass, and sleeve gastrectomy procedures, in comparison with standard nonsurgical health care management of obesity. We also aimed to determine whether there were population subgroups defined by age, sex, socioeconomic position, obesity category, and comorbidity for whom bariatric surgery might be more, or less, cost-effective.

## Methods

### Model Structure

**Fig. 1:**
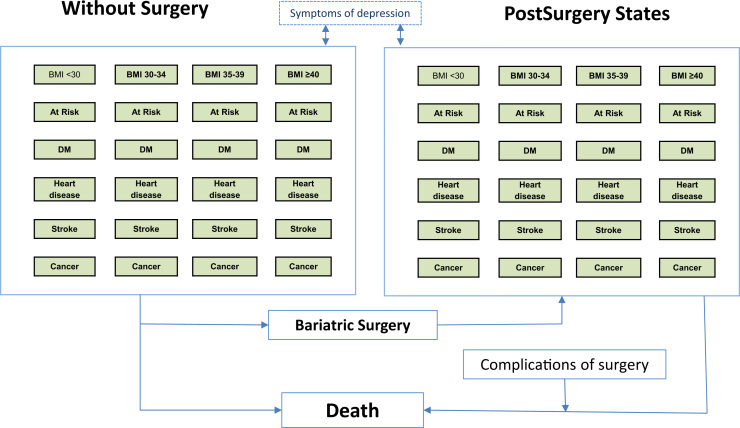
Structure of the Markov model. BMI, body mass index; DM, diabetes mellitus.

A Markov model was used to conduct a cost-utility analysis to compare a strategy in which all eligible participants received bariatric surgery, with standard nonsurgical weight management. Because the use of different bariatric surgical procedures has changed rapidly in recent years [9], bariatric surgery was assumed to comprise one-third each of three main procedures—gastric banding, gastric bypass, and sleeve gastrectomy. We did not aim to explore the comparative effectiveness of different bariatric surgical procedures within the scope of this research. The model structure (Fig. 1) included the main influences on costs and outcomes of bariatric surgery (see Table 1 in Supplemental Materials found at doi:10.1016/j.jval.2016.08.734). Healthy subjects, referred to as “At Risk,” may develop one of the disease states of interest, including diabetes mellitus, coronary heart disease (CHD), stroke, or cancer. Participants in each state were allowed to progress to depression, with each main state divided into substates representing “not depressed” and “depressed” [18]. The model was stratified by body mass index (BMI) category, comprising morbid obesity, severe obesity, simple obesity, overweight, and normal weight, and allowed participants to transition between BMI categories.

In each BMI category, there were five disease states that were divided into “depressed” or “not depressed” (Fig. 1). All states might lead to death. There were 101 states included in the model across two treatment conditions. Each state was further stratified by single year of age and sex. The perspective of the model was that of health care services and only health care costs were included. A lifetime time horizon was used.

### Cohort Study Using Electronic Health Records

Data to populate the model were derived by an epidemiological analysis of electronic health records data from two cohorts of participants drawn from the general population registered with the UK Clinical Practice Research Datalink (CPRD). The CPRD includes electronic health records of participants registered with a nationally representative sample of approximately 600 UK family practices.

Estimates for the incidence, prevalence, mortality, and costs of health care utilization for each state in the model were obtained from data for a cohort of 247,537 participants drawn from CPRD who did not receive bariatric surgery. The cohort was sampled from the May 2014 release of CPRD and included patients who were registered with CPRD practices between January 1, 2008, and December 31, 2013. A random sample of up to 50,000 participants was sampled from each BMI category including 18.5 to 25, 25 to 29, 30 to 34, 35 to 39, 40 to 44, and 45 kg/m^2^ or higher. The last two categories were combined for analysis so as to treat morbid obesity as a single category. Estimates by deprivation quintile were obtained for participants with Indices of Multiple Deprivation 2010 (IMD 2010) quintile data linked through patients’ postcodes. This deprivation index score is a nationally produced summary measure of deprivation constructed from a weighted combination of metrics for deprivation on the domains of income, employment, health, education, access to services, environment, and crime. These represent a broad range of social and material deprivations [19]. Incidence and mortality rates were estimated in a time-to-event framework using a Weibull model (see Table 2 in Supplemental Materials found at doi:10.1016/j.jval.2016.08.734). The start of the analysis was the date on which the practice joined CPRD or the date on which the patient joined a CPRD general practice if this was later; the end of the analysis was at the end of the patient’s record in CPRD, or the death date or the comorbidity start date if these were earlier. For analysis of incidence, covariates were age, age-squared, sex, and BMI category. For analysis of mortality, covariates also included each comorbidity category, including diabetes, CHD, stroke, and cancer. This facilitated the estimation of mortality probabilities for each comorbidity category. The median duration of analysis time was 5.6 years (interquartile range, 2.3–10.3 years). The prevalence of depression was estimated from CPRD data for each state in the model [18]. Mortality was assumed to be independent of depression status. Health care utilization was estimated for each state from CPRD records with linked hospital episode statistics data, including use of primary care (family practice consultations, telephone consultations, home visits, and emergency and out-of-hours consultations), secondary care (including hospital admissions, outpatient visits, day case visits, and emergency visits), and prescriptions as reported elsewhere [20]. The annual costs associated with each state were estimated by multiplying the health care utilization associated with the state by the costs of each unit of health care, which were obtained from standard reference sources for 2013 [21] (see Table 3 in Supplemental Materials found at doi:10.1016/j.jval.2016.08.734). Costs for each prescription record were obtained from RESIP UK (Chertsey, Surrey, UK). Patient-level costs were estimated from CPRD records using a two-part regression model as reported previously [18], [20]. A cohort of participants who underwent bariatric surgery was also drawn from CPRD and used to evaluate baseline use of bariatric surgery and associations with diabetes incidence, diabetes remission, and depression prevalence as reported previously [5], [22].

### Model Estimation

The probabilistic Markov model was estimated by cohort simulation, implemented through a program written in R software (R Foundation for Statistical Computing, Vienna, Austria) [23]. The initial population had ages ranging from 20 to 74 years, and we observed that there were few bariatric surgical procedures for those older than 74 years in CPRD. The proportion of the start population with morbidity was also informed by an analysis of a CPRD cohort.

All simulations were stratified by single year of age with the initial population aging by 1 year per cycle. Participants exited the model when they reached 100 years of age or died. Annual transition probabilities for the model were obtained by sampling from the beta-binomial distribution, using CPRD data as inputs. The costs associated with each state were sampled from the gamma distribution with the predicted mean value estimated from a two-part model as outlined earlier. Utilities for each state were obtained from data published in a compendium of values [24] (see Table 4 in Supplemental Materials found at doi:10.1016/j.jval.2016.08.734). Utility values for each state were stratified by single year of age but were the same for men and women. Utility values were sampled from the beta distribution. Total costs and quality-adjusted life-years (QALYs) were obtained by summing across the 81 cycles of the model included in each simulation. Results are expressed as rates per 1,000 participants entering the model. Mean costs and the 95% CI were obtained from the data for 1000 simulations. Costs and QALYs were discounted using a rate of 3.5%, but undiscounted values and values discounted at 1.5% are also shown as sensitivity analyses [25]. Net monetary benefits (NMBs) and net health benefits (NHBs) were estimated at threshold values of £20,000 and £30,000 per QALY, respectively [25].

### Intervention Effects and Costs of Bariatric Surgery

The effect of bariatric surgery was modeled as a reduction in disease incidence and mortality (Table 1). The effect of bariatric surgery on the incidence of diabetes was drawn from CPRD data analyses [5] that gave results very similar to data from the Swedish Obese Subjects Study [7]. Effects on incidence of CHD, stroke, and cancer were also drawn from the Swedish Obese Subjects Study [7], [26], which showed a reduction in cancer incidence in women but not in men [26]. The relative risk of mortality after bariatric surgery was obtained from Arterburn et al. [8] and this relative risk was applied to the estimation of mortality probabilities in each model state. The effect on depression prevalence was drawn from CPRD data analyses [22] and is also consistent with other reports. On the basis of CPRD data analyses, 40% of patients with diabetes entered remission after the procedure [27]. Bariatric surgery was modeled as being associated with a positive impact on patient utility of equal weight to a two-unit change in BMI category [4], [28]. This effect, however, was modeled to decline over time, according to year to the power −0.25, consistent with the known reduction in the initial quality-of-life improvement after bariatric surgery [29]. The costs of bariatric surgery were drawn from National Health Service tariffs and included preoperative weight management, the cost of the procedure, and postoperative reviews (Table 1). Bariatric surgery was assumed to comprise one-third each of gastric banding, gastric bypass, and sleeve gastrectomy. The cost of leaks was included as an average cost across all patients [30]. Two percent of patients were assumed to require repeat procedures each year, slightly higher than the 1.2% observed in CPRD records [9]. Mortality from surgery was estimated at 0.07% from the National Bariatric Surgical Register report [14]. Costs of health care utilization were estimated from CPRD (see Table 4 in Supplemental Materials). Costs of health care utilization after bariatric surgery were determined by age, sex, and morbidity category, and were not modeled as associated with body weight reduction, consistent with the results of empirical studies [10], [11].

### Subgroup and Sensitivity Analyses

Subgroup analyses were performed to estimate costs and outcomes separately for men and women, for separate age groups and categories of deprivation, comparing the most- and the least -deprived quintiles of deprivation. Sensitivity analyses were performed to explore the effects of varying the unit costs of bariatric surgery, including values 50% and 100% higher than the base case, and of varying the discount rate including values of 0%, 1.5%, and 3.5%. These were conducted to also estimate outcomes assuming that intervention effects after bariatric surgery might diminish with time. This was implemented by allowing intervention effects from bariatric surgery to diminish by year to the power −0.25 or −0.50. The former implies that the effect of bariatric surgery will decline by 44% over 10 years, whereas the latter indicates that the intervention effect will decline by 68% over 10 years. This is in addition to the modeled time-related decline in utility benefit from bariatric surgery described earlier. A sensitivity analysis was also used to test intervention cost-effectiveness for patients with severe obesity (BMI 35–39 kg/m^2^) or with morbid obesity and diabetes.

The use of fully anonymized CPRD data was approved by the Medicines and Healthcare products Regulatory Agency’s Independent Scientific Advisory Committee (Protocol number 13_089).

Protocol: The protocol for the study has been published online at http://www.nets.nihr.ac.uk/projects/hsdr/12500512 and http://www.nets.nihr.ac.uk/__data/assets/pdf_file/0005/81806/PRO-12-5005-12.pdf.

## Results

The population entering the Markov model comprised 200,000 participants with a BMI of 40 kg/m^2^ or higher with equal numbers of men and women (mean age 46 years; range 20–74 years). There were 19% with diabetes mellitus and 4% with CHD, the remaining having no chronic comorbidity (Table 2).

Bariatric surgery was associated with an increase in total life-years, accumulated over the lifetime of participants entering the model, of 6,097 per 1,000 participants entering the model (Table 2). There was a substantial increase in the number of life-years lived free from chronic comorbidities of 10,297 per 1,000. There was a decrease in life-years lived with diabetes mellitus of 8,320 per 1,000. There were modest increases in life-years lived with CHD, stroke, and cancer after bariatric surgery because of the increase in the population at risk for these conditions over the lifetime of the model.

The total undiscounted health care costs over a lifetime for 1,000 persons with morbid obesity were estimated to be £97.82 million in the absence of bariatric surgery and £126.84 million with bariatric surgery (Table 3). The undiscounted incremental cost associated with bariatric surgery was £29.01 million, or £15.26 million when discounted at 3.5%. The cost of the bariatric surgical procedure is estimated to be £9.16 million per 1,000 participants, and so it can be concluded that bariatric surgery is associated with increased lifetime health care costs associated with greater longevity. This is reflected in the greater estimated discounted QALYs after bariatric surgery of 14,509 per 1,000 persons compared with 12,367 in the absence of bariatric surgery. The net gain in discounted QALYs from bariatric surgery was 2,142 per 1,000 persons. The estimated value for discounted cost per QALY gained was £7,129 per QALY, with a 95% CI from 1,000 simulations of £6,774 to £7,506 per QALY. If each QALY gained is valued at £30,000, then the net benefit associated with bariatric surgery performed in 1,000 persons with morbid obesity is approximately £49 million, or £28 million if a value of £20,000 per QALY is used.

Table 4 presents the results of the analyses in subgroups of the population as well as those of the sensitivity analyses that varied underlying assumptions. All estimates were discounted at 3.5%. Incremental costs and QALYs were slightly lower in men than in women, reflecting the general lower life expectancy in men, but estimates of costs per QALY were similar in men and women. Older participants generally incurred lower total costs and fewer total QALYs, consistent with their shorter life expectancy, but incremental costs and QALYs were higher as a result of the higher absolute risk reductions obtained in a population at higher baseline risk. Nevertheless, cost-effectiveness estimates were generally consistent across age groups. Comparing the use of bariatric surgery in the most and the least-deprived quintiles of deprivation, total costs were higher and total QALYs were lower in the most-deprived quintile but incremental costs and QALYs were similar in each deprivation category as were cost-effectiveness estimates. The model was run using an initial population with severe obesity and the procedure was found to be only slightly less cost-effective in this group with an estimated cost of £7,675 per QALY. When the initial population was confined to morbidly obese persons with diabetes, the estimated cost per QALY was £6,176 (£5,894–£6,457).

Estimates of cost-effectiveness were sensitive to the cost of the surgical procedure. Nevertheless, even when the cost of the procedure was 100% higher than that in the base case (£18,328 instead of £9,164), bariatric surgery was cost-effective at £11,376 per QALY. Simulations in which the procedure cost was set at 0 confirmed that incremental health care costs remained positive. When the intervention effect from bariatric surgery was allowed to diminish markedly over time after the procedure, there was only a modest impact on estimated cost-effectiveness (Table 4), reflecting the smaller contribution made by discounted costs and QALYs from later periods of follow-up.

Table 5 presents the estimates for a health care commissioning organization with a population of 250,000. On the basis of prevalence rates for England, there may be 7,163 individuals with morbid obesity, of whom 1,406 may have diabetes. The cost of 1,000 bariatric surgical procedures will be £9,164 million. Over the lifetime of patients who undergo bariatric surgery, the total increase in health care costs may be £15.26 million. If these procedures are divided equally among patients with diabetes and those without diabetes, there will be 112 fewer patients diagnosed with diabetes over 10 years and at least 200 patients with diabetes may enter remission. The health gain amounts to 2,142 QALYs and, valued at £30,000 per QALY, NHBs are expected to be £49 million.

## Discussion

### Main Findings

This research modeled the lifetime health benefits and health care costs from bariatric surgery. The results project substantial increases in life-years and reductions in years lived with diabetes. Bariatric surgery is expected to be associated with increased health care costs arising from the costs of the procedure, as well as increased lifetime health care costs associated with increased life expectancy. When health benefits and costs are combined into a single metric, using accepted values of cost per QALY, the use of bariatric surgery is expected to yield substantial NMBs amounting, over a lifetime, to £49 million per 1,000 persons. NHBs, after allowing for additional costs, may amount to 1,634 QALYs per 1,000 persons. Bariatric surgery has similar cost-effectiveness in men and in women, at different ages and in different deprivation categories. Bariatric surgery is also expected to be cost-effective in individuals with severe obesity (BMI 35–39 kg/m^2^). Bariatric surgery will be cost-effective even if the cost of the procedure is twice as high as we have estimated, or if the effect of the procedure declines over time so that only 32% of the initial effect remains after 10 years.

This research made a comparison between “bariatric surgery” and “no bariatric surgery” as an intervention for severe obesity. There are several bariatric surgical procedures in use at present. Over the last 10 years there has been a dramatic reduction in the use of adjustable gastric banding procedures, with an increase in the use of gastric bypass and sleeve gastrectomy procedures [9]. Although gastric banding is associated with lower procedure costs, there may be a higher rate of revisional surgery [9]. Gastric banding also has smaller, and less well-maintained, effects on body weight [3] and diabetes outcomes [27] than do gastric bypass or sleeve gastrectomy procedures. Future research is required to evaluate the comparative cost-effectiveness of different bariatric surgical procedures. This will require improved long-term outcome data for procedures that are presently in use. At present, some of the longest follow-up data are from the Swedish Obese Subjects Study in which most of the participants underwent the vertical banded gastroplasty procedure, which is now less used. Our sensitivity analyses incorporated the possibility that in future bariatric surgery may be more costly than it is at present, as would be the case if a higher number of gastric bypass or sleeve gastrectomy procedures were performed, or that the intervention effect decayed rapidly over time, or if a higher number of banding procedures were performed. In either eventuality, bariatric surgery would remain highly cost-effective.

### Comparison with Previous Studies

There have been several previous cost-effectiveness analyses of bariatric surgery for morbid obesity. One of the most authoritative was the 2009 study by Picot et al. [31]. Their study reported a model in which weight loss after surgery was viewed as the main mediator of longer term changes in health outcomes. The study found that bariatric surgery was a cost-effective intervention for morbid obesity, with incremental cost-effectiveness ratios ranging between £2,000 and £4,000 per QALY gained over a 20-year time horizon [31]. A more recent study suggested that bariatric surgery may be cost saving for health care systems through reduced morbidity [16], but this conclusion has been disputed. Although bariatric surgery does not appear to generate cost savings, its use is associated with substantial health gains at costs that are well less than accepted thresholds for cost-effectiveness.

This analysis updates previous studies by including those surgical procedures that are presently used, including adjustable gastric banding, gastric bypass, and sleeve gastrectomy. The analysis also updated the costs of surgery to present-day values. The present model incorporated direct evidence concerning the long-term outcomes of bariatric surgery in contrast to previous studies, which have used changes in intermediate measures of surgical effect including body weight, blood pressure, and lipid profiles to model the long-term outcomes of bariatric surgery. Our analysis recognized that the effects of surgery, particularly those on diabetes, are not entirely weight-dependent and our model therefore did not include weight change as an intermediate outcome. The model was informed by recent measures of the effects of surgery on substantive long-term health outcomes and health care costs, additionally informed by analysis of CPRD electronic health records. In spite of this difference of approach, our analyses are consistent with previous reports in showing that bariatric surgery is likely to be very cost-effective. We do not find that bariatric surgery is cost saving. This would, however, not be expected because it is unlikely for a procedure that reduces mortality in a population that experiences a heavy burden of morbidity to reduce lifetime health expenditures [17].

### Study Limitations

This research was based on empirical data for disease incidence, mortality, and costs of health care utilization estimated from the electronic health records of a large sample of participants managed in primary care in the United Kingdom between 2008 and 2013. The study drew on recently published and authoritative estimates of the effects and costs of bariatric surgery with an emphasis on those bariatric surgical procedures that are presently used. We have used conservative assumptions including that costs of health care utilization after surgery are not associated with weight loss; that any gain in utility associated with BMI reduction declines rapidly over time; and that remission from diabetes after surgery occurs in 40% of patients, as estimated from CPRD, which is lower than levels observed in other reported studies. We acknowledge that any model represents a simplification of reality. There are other forms of morbidity that were not represented in the model. Nevertheless, the costs of health care utilization from such conditions will have been included in cost estimates from CPRD, which encompassed all health care utilization. We included the major complications of surgery including operative mortality, costs of leaks after surgery, and re-operations in 2% of patients per year. There may be additional costs associated with surgery but a sensitivity analysis showed that even if total costs of surgery were to be twice as high as estimated in the base case, bariatric surgery would still be cost-effective. We modeled bariatric surgery as having a constant effect in the postoperative period, but we showed that even if the effects declined over time, the surgery would still be cost-effective. The model adopted a lifetime perspective by using age-specific estimates from the present time. The large sample size yielded precise estimates even for advanced ages. We acknowledge that age-specific mortality rates in future are likely to be different from those observed today. We note that it is possible that bariatric surgery may result in “spillover” effects or positive externalities if benefits are transmitted to other family members. For example, lifestyle changes on the part of a mother might have an impact on the obesity risk and health behaviors of the entire family. These effects might be quantified and modeled in future research.

## Conclusions

Bariatric surgery is highly cost-effective and substantial NHBs or NMBs may be anticipated from wider use of bariatric surgical procedures in patients with severe and morbid obesity. Similar cost-effectiveness may be anticipated in diverse groups of obese individuals including men and women, wide ranges of ages, and different levels of deprivation. Morbid obesity shows strong socioeconomic patterning, and consequently bariatric surgery may have the potential to reduce obesity-related inequalities in health if there is equitable patient selection. This is in contrast to presently available nonsurgical interventions for obesity, which generally have only small and short-lived effects [32]. On the basis of these results, increasing access to bariatric surgery may be a justifiable choice for publicly funded health care systems but cost savings are not anticipated. The potential demand for bariatric surgery is, however, likely to greatly exceed plausible levels of supply. A major concern remains that the social and environmental drivers of the increase in morbid obesity should not remain unchecked. As a “downstream” procedure, bariatric surgery will not stem the global increase in obesity. The role of surgery in treating disorders apparently rooted in individual lifestyle is also questioned. There are also concerns relating to the capacity of health services to deliver safe, high-quality services for patients with severe and morbid obesity, which were recognized in the national guidance on bariatric surgery. Nevertheless, bariatric surgery may often offer important health gains at an acceptable level of investment.

Source of financial support: This research was supported by the UK National Institutes for Health Research (NIHR) Health Services and Delivery Research program. The funders did not engage in the design and conduct of the study; collection, management, analysis, and interpretation of the data; and preparation, review, or approval of the manuscript. T. Prevost and M.C. Gulliford are supported by the NIHR Biomedical Research Centre at Guy’s and St Thomas’ National Health Service Foundation Trust and King’s College London. The views expressed are those of the authors and not necessarily those of the National Health Service, the NIHR, or the Department of Health.

## Figures and Tables

**Table 1:** Data for costs and effects of bariatric surgery

**Item**	**Value**	**Source**
Preoperative weight management	£1024	Tier 3 weight management program
Procedure cost	£7015	NHS tariff: LAGB £3620; GBP £8713; sleeve gastrectomy £8713 (33% each procedure)
Postoperative review	£875	
Cost of leaks	£250	Bransen et al. [30]
Total procedure cost	£9164	
Cost of re-operations	£3620	NHS tariff (FZ05A)
Rate of re-operation	2% per year	CPRD (unpublished analyses)

Operative mortality	0.07%	NBSR [14]

Diabetes remission	40%	CPRD [25]

Incidence (relative risk)		
Diabetes mellitus	0.20 (0.13–0.30)	CPRD [5]
CHD	0.67 (0.54–0.83)	SOS [7]
Stroke	0.67 (0.54–0.83)	SOS [7]
Cancer	0.58 (0.44–0.77)	SOS [26] (women only)

Mortality (relative risk)	0.45 (0.36–0.56)	Arterburn et al. [8]

Depression (relative risk)		
Year 1	0.82 (0.78–0.87)	CPRD [22]
Year 2	0.83 (0.76–0.90)	
Year 3	0.87 (078–0.97)	

Decrement in utility associated with BMI (kg/m^2^) category		
25–29	0	Hakim et al. [28]
30–34	−0.085	
35–39	−0.17	
≥40	−0.255	

BMI, body mass index; CHD, coronary heart disease; CPRD, Clinical Practice Research Datalink; GBP, gastric bypass; LAGB, laparoscopic adjustable gastric banding; NBSR, National Bariatric Surgical Register; NHS, National Health Service; SOS, Swedish Obese Subjects Study.

**Table 2:** Distribution of initial population and model outputs per 1,000 participants entering the model

**Measure**	**Bariatric surgery**	**No bariatric surgery**	**Incremental value**	
			**Mean**	**95% CI**
*Initial population entering the model*				
Number	200,000	200,000	–	
Age (y), mean (range)	46 (20–74)	46 (20–74)	–	

Men	100,000	100,000	–	
Women	100,000	100,000	–	

No morbidity	153,846 (77)	153,846 (77)	–	
Diabetes mellitus	38,462 (19)	38,462 (19)	–	
CHD	7,692 (4)	7,692 (4)	–	

*Model outputs (rate per 1,000 persons entering the model)*				
Total person-years lived	41,869.28	35,772.21	6,097	6,022 to 6,171
No morbidity (person-years)	22,296.44	11,998.61	10,297	10,152 to 10,452
Diabetes mellitus (person-years)	9,434.01	17,754.62	−8,320	−8,502 to −8,123
CHD (person-years)	5,321.58	3,771.50	1,550	1,473 to 1,626
Stroke (person-years)	1,309.54	633.92	676	647 to 705
Cancer (person-years)	3,507.70	1,613.56	1,894	1,830 to 1,957
Depression (person-years)	4,393.11	4,385.58	8	−8 to 23

*Note*. Figures are frequencies (column percent) except where indicated.

CHD, coronary heart disease; CI, confidence interval.

**Table 3:** Cost-utility analysis of bariatric surgery in morbid obesity

**Measure**	**Bariatric surgery**	**No bariatric surgery**	**Incremental value**	
			**Mean**	**95% CI**
*Health care costs per 1,000 (£, millions)*				
Not discounted	126.84	97.82	29.01	28.78–29.23
Discounted 1.5%	93.06	72.38	20.68	20.53–20.81
Discounted 3.5%	67.25	51.99	15.26	15.18–15.33

*QALYs per 1,000*				
Not discounted	28,345	22,772	5,572	5,422–5,728
Discounted 1.5%	20,547	17,022	3,524	3,397–3,655
Discounted 3.5%	14,509	12,367	2,142	2,032–2,256

*Cost (£) per QALY*				
Not discounted			5,208	5,075–5,338
Discounted 1.5%			5,868	5,662–6,073
Discounted 3.5%			7,129	6,775–7,506

*NMB per 1,000 (£, millions)*				
£30,000 per QALY			49.02	45.72–52.41
£20,000 per QALY			27.59	25.40–29.85

*NHB per 1,000 (QALYs)*				
£30,000 per QALY			1,634	1,524–1,747
£20,000 per QALY			1,380	1,270–1,493

NHB, net health benefit; NMB, net monetary benefit; QALY, quality-adjusted life-year.

**Table 4:** Cost-utility analyses for subgroup and sensitivity analyses

**Condition**	**Bariatric surgery arm**		**Incremental**		**Cost per QALY**	
					**Mean**	**95% CI**
	**Total costs (£, millions)**	**Total QALY**	**Incremental costs (£, millions)**	**Incremental QALY**		
BMI ≥ 40	67.25	14,509	15.26	2,142	7,129	6,775–7,506
35–39	68.08	14,708	15.00	1,995	7,675	7,339–8,037
Sex						
Male	63.99	14,332	14.97	2,087	7,188	6,662–7,796
Female	70.51	14,680	15.55	2,201	7,076	6,581–7,638
Age group (y)						
20–34	68.18	17,153	13.62	1,866	7,344	6,478–8,421
35–54	70.79	15,030	15.00	2,139	7,027	6,511–7,569
55–74	59.49	11,545	17.01	2,355	7,230	6,862–7,613
Deprivation category						
Least deprived	61.49	14,791	14.46	2,052	7,056	6,688–7,448
Most deprived	70.00	14,187	16.32	2,242	7,287	6,930–7,665
Diabetes BMI						
≥40	68.47	14,468	15.04	2,437	6,176	5,894–6,457
Costs of procedure						
50% higher	71.83	14,511	19.84	2,144	9,261	8,800–9,795
100% higher	76.41	14,512	24.42	2,148	11,376	10,763–11,950
Zero procedure cost	58.09	14,512	6.10	2,148	2,842	2,701–2,998
Decline of intervention effect over time						
Year^−0.25^	64.25	13,786	12.25	1,422	8,637	8,009–9,400
Year^−0.50^	63.15	13,516	11.16	1,152	9,720	8,860–10,706

*Note*. Figures are expressed as rates per 1,000 persons entering the model except where indicated.

BMI, body mass index; QALY, quality-adjusted life-year.

**Table 5:** Implications of research for an HCO with a population of 250,000

**Measure**	**Estimate**	**Source**
Total population aged 20–74 y	250,000	
Number with morbid obesity	7,163 (4,875 women and 2,288 men)	Prevalence of obesity from Health Survey for England 2011–2013
Number with morbid obesity and diabetes	1,406 (763 women and 643 men)	Prevalence of diabetes by BMI category from Health Survey for England 2011–2013
Cost of 1,000 bariatric surgical procedures, with 50% in people with diabetes	£9.164 million	Table 1
Total increase in health care costs over patients’ lifetime	£15.260 million	Table 4
Number of new cases of diabetes prevented over 10 y	112	[5]
Number of diabetes cases in remission over next 5 y	200	[25]
Health gain in QALYs over patients’ lifetime	2,142	Table 4
NMBs over patients’ lifetime (£30,000 per QALY)	£49 million	Table 4
Number with morbid obesity if the HCO is among the most deprived	10,813 (7,663 women and 3,150 men)	Prevalence of obesity by deprivation quintile from Health Survey for England 2011–2013 combined
Number with morbid obesity if the HCO is among the least deprived	4,413 (2,838 women and 1,575 men)	

BMI, body mass index; HCO, health care commissioning organization; NMBs, net monetary benefits; QALY, quality-adjusted life-year.
