# Supplementary material for: Costs and Outcomes of Increasing Access to Bariatric Surgery: Cohort Study and Cost-Effectiveness Analysis Using Electronic Health Records
Source: Value Health. 2017 Jan;20(1):85–92. doi: 10.1016/j.jval.2016.08.734 (PMC5338873; doi:10.1016/j.jval.2016.08.734)
Supplement: Supplementary file 1 — Supplementary material [file mmc1.docx]

**Access to bariatric surgery**

**Supplementary Table 1: Variables associated with cost-effectiveness of bariatric surgery.**

| **Variable** | **Incorporated into model as:** |
| --- | --- |
|  |  |
| **Favours bariatric surgery** |  |
| Reduced incidence of disease | Relative risks associated with bariatric surgery from CPRD and SOS (5-7) (Table 1) |
| Reduced mortality | Relative risks associated with bariatric surgery from previous reports (8) (Table 1) |
| Transient reduction in depression | Relative risks associated with bariatric surgery from CPRD (20) (Table 1) |
| Greater utility associated with lower morbidity | Utility values for each model state from previous reports (22) (Supplementary Table 3) |
| Increase in utility associated with BMI reduction | Time-dependent utility increment associated with surgery (25,26) (Table 1) |
| Reduced costs of health care utilisation resulting from lower morbidity | Costs of each state estimated by year of age and gender from CPRD data (Supplementary Table 4) |
|  |  |
| **Favours no surgery** |  |
| Cost of surgery | Costs of surgery estimated from NHS tariffs (Table 1) |
| Operative mortality | Mortality from NBSR report (14) (Table 1) |
| Complications following surgery | Costs of complications including leaks and re-operations (Table 1) |
| Increased health care costs from greater longevity | Costs of each state estimated by year of age from CPRD data (Supplementary Table 4) |
|  |  |

CPRD, Clinical Practice Research Datalink; NBSR, National Bariatric Surgical Register; NHS, National Health Service; SOS, Swedish Obese Subjects study

**Supplementary Table 2. Regression coefficients associating body mass index category with incidence and mortality. (‘Overweight’ was employed as reference BMI category; ‘At Risk’ was employed as reference morbidity category. Please see text for further explanation)**

|  |  | **Gender** | **Age** | **Age^2^** | **Normal weight** | **Simple Obesity** | **Severe obesity** | **Morbid obesity** | **Constant** | **Gamma** |
| --- | --- | --- | --- | --- | --- | --- | --- | --- | --- | --- |
|  |  |  |  |  |  |  |  |  |  |  |
| *Incidence of morbidity* | |  |  |  |  |  |  |  |  |  |
| Diabetes mellitus | | -0.34 | 0.05 | -0.00 | -0.86 | 0.78 | 1.32 | 2.00 | -6.74 | -0.10 |
| CHD |  | -0.70 | 0.22 | -0.00 | -0.25 | 0.10 | 0.08 | 0.26 | -12.0 | -0.06 |
| Stroke |  | -0.40 | 0.09 | -0.00 | -0.10 | -0.01 | 0.03 | 0.25 | -11.0 | 0.14 |
| Cancer | Male | - | 0.19 | -0.00 | 0.12 | -0.14 | -0.34 | -0.25 | -12.4 | 0.18 |
| Cancer | Female | - | 0.15 | -0.00 | 0.12 | -0.05 | -0.07 | 0.11 | -10.8 | 0.15 |
| *Mortality^a^* |  | -0.31 | 0.03 | 0.00 | 0.37 | -0.01 | 0.16 | 0.74 | -10.4 | 0.30 |
|  |  |  |  |  |  |  |  |  |  |  |

^a^in the analysis of mortality, coefficients for comorbidity were DM 0.44; CHD 0.24; Stroke 0.83; Cancer 1.65

**Supplementary Table 3: Unit Costs of health care utilisation. Source: Reference 19.**

| **Type of care** | **Unit Cost 2013** | **Comment** |
| --- | --- | --- |
|  |  |  |
|  |  |  |
| GP consultations | £45 | Includes emergency consultations |
| Telephone consultations | £27 |  |
| Home visits | £114 |  |
| Day Case | £697 |  |
| Emergency referral | £135 | From outpatient |
| Inpatient | £1,400 | Weighted average of all stays |
| Outpatient | £135 | Weighted average of all outpatient |
|  |  |  |

**Supplementary Table 4. Utility values used in model. Source: reference 24.**

| **Condition** |  | **Utility** |
| --- | --- | --- |
|  |  |  |
| Mean adult utility at age 43 | | 0.828 |
| Age (per year increase) | | -0.00029 |
| Diabetes mellitus |  | -0.0714 |
| Coronary heart disease | | -0.0671 |
| Stroke |  | -0.1171 |
| Cancer |  | -0.04347 |
| Depression |  | -0.1123 |
|  |  |  |
| BMI category (Kg/m^2^) | 30-34 | -0.085 |
|  | 35-39 | -0.17 |
|  | 40+ | -0.255 |
|  |  |  |
